# Improving the MVA Vaccine Potential by Deleting the Viral Gene Coding for the IL-18 Binding Protein

**DOI:** 10.1371/journal.pone.0032220

**Published:** 2012-02-22

**Authors:** Juliana Falivene, María Paula Del Médico Zajac, María Fernanda Pascutti, Ana María Rodríguez, Cynthia Maeto, Beatriz Perdiguero, Carmen E. Gómez, Mariano Esteban, Gabriela Calamante, María Magdalena Gherardi

**Affiliations:** 1 Centro Nacional de Referencia para el SIDA, Facultad de Medicina, Universidad de Buenos Aires, Buenos Aires, Argentina; 2 Instituto de Biotecnología, CICVyA-INTA Castelar, Buenos Aires, Argentina; 3 Departamento de Biología Molecular y Celular, Centro Nacional de Biotecnología, CSIC, Campus Universidad Autónoma, Madrid, Spain; Imperial College London, United Kingdom

## Abstract

**Background:**

Modified Vaccinia Ankara (MVA) is an attenuated strain of Vaccinia virus (VACV) currently employed in many clinical trials against HIV/AIDS and other diseases. MVA still retains genes involved in host immune response evasion, enabling its optimization by removing some of them. The aim of this study was to evaluate cellular immune responses (CIR) induced by an IL-18 binding protein gene (*C12L*) deleted vector (MVAΔC12L).

**Methodology/Principal Findings:**

BALB/c and C57BL/6 mice were immunized with different doses of MVAΔC12L or MVA wild type (MVAwt), then CIR to VACV epitopes in immunogenic proteins were evaluated in spleen and draining lymph nodes at acute and memory phases (7 and 40 days post-immunization respectively). Compared with parental MVAwt, MVAΔC12L immunization induced a significant increase of two to three-fold in CD8^+^ and CD4^+^ T-cell responses to different VACV epitopes, with increased percentage of anti-VACV cytotoxic CD8^+^ T-cells (CD107a/b^+^) during the acute phase of the response. Importantly, the immunogenicity enhancement was also observed after MVAΔC12L inoculation with different viral doses and by distinct routes (systemic and mucosal). Potentiation of MVA's CIR was also observed during the memory phase, in correlation with a higher protection against an intranasal challenge with VACV WR. Of note, we could also show a significant increase in the CIR against HIV antigens such as Env, Gag, Pol and Nef from different subtypes expressed from two recombinants of MVAΔC12L during heterologous DNA prime/MVA boost vaccination regimens.

**Conclusions/Significance:**

This study demonstrates the relevance of IL-18 bp contribution in the immune response evasion during MVA infection. Our findings clearly show that the deletion of the viral IL-18 bp gene is an effective approach to increase MVA vaccine efficacy, as immunogenicity improvements were observed against vector antigens and more importantly to HIV antigens.

## Introduction

Modified Vaccinia virus Ankara (MVA), an attenuated strain of Vaccinia virus, was obtained following extensive serial passages on primary chicken embryo fibroblasts (CEFs) [1]. During this process of attenuation, MVA underwent deletion of 31 kbp (15%) of its genome, as compared to its parental strain, including a number of genes that contribute to viral evasion from host immune responses and that determine virus host range [2], [3]. As a result, MVA lost its ability to replicate in most mammalian cells, including primary human cells [4], [5]. However, MVA has conserved the characteristic ability to induce robust T-cell immune responses against recombinant antigens, comparable to those generated by more virulent replication competent VACV strains [6], [7], [8]. Its safety as a vaccine vector has been largely proved during the vaccination of more than 100.000 individuals against smallpox without side effects [9]. Thus, the highly advantageous safety characteristics showed by MVA, in addition to its ability to express high levels and numbers of foreign genes, has converted it as one of the leading candidates for evaluation as a vaccine vector in multiple human clinical trials against different infection diseases [10], [11], [12] and also melanoma [13].

Despite its large loss of genomic regions during the attenuation process, MVA still retains viral genes involved in host immune response evasion, raising the possibility to increase its vaccine potential by removing some of them. Examples of this test of concept have been recently shown in the literature, as the enhancement of MVA immunogenicity after the removal of the gene that encodes an interleukin 1β (IL-1β)-binding protein that is secreted from infected cells [14]; or the increment of its vaccine efficacy after the removal of the gene *A41L* that encodes for a chemokine-binding protein [15]; or removal of the gene *C6L* that encodes an inhibitor of IFN-β induction [16]. Another gene with immunomodulatory properties that has been conserved in the MVA genome is the *008L* gene (*C12L* in VACV) that codes for an interleukin 18 binding protein (IL-18 bp) [17], [18]. IL-18 bps have been described in humans and mouse as soluble inhibitors that bind and neutralize endogenous IL-18 [19]. IL-18 has important roles in the regulation of both innate and specific immune responses. This cytokine is an important mediator in the Th1 response, primarily by induction of IFN-γ secretion from T-cells and natural killer (NK) cells [20], it also enhances T and NK cell maturation, cytokine production, and cytotoxicity [21], [22], [23]. Furthermore, IL-12 and IL-18 act synergistically to promote Th1-mediated immune responses, which play a critical role in defense against intracellular microbes through the production of IFN-γ [21].

Past reports have firstly described that the orthopoxviruses VACV, ectromelia virus (EV), and cowpox virus express a soluble IL-18 bp (vIL-18 bp), encoded by homologs of the variola virus D7L ORF that is secreted from infected cells [17], [24]. Expression of this immunomodulator by distinct poxvirus strains emphasizes the importance of IL-18 in the course of viral infections as immune evasion mechanisms. The *C12L* gene of the VACV Western Reserve (WR) strain was previously characterized in BALB/c mice. Results showed that after inoculation of mice by intranasal (i.n) route, a deletion mutant for this gene was attenuated and induced lower weight loss and signs of illness compared to controls [18]. Afterwards, the same authors performed a more in depth study in which they demonstrated a role for the vIL-18 bp in counteracting IL-18 in both the innate and the specific immune response to VACV infection, highlighting the ability of IL-18 to promote vigorous antiviral T-cell responses [25]. A more recent study described the effects of the deletion of the IL-18 bp gene from the genome of another replicating VACV strain, the Tiantan Vaccinia virus (TV) vector, in which the deletion diminished the virulence of the parental virus while immunogenicity was not affected [26].

Although the studies in which the deletion of IL-18 bp coding gene from the VACV WR genome documented an improvement in the cellular immunity induced by the deletion mutant, in relation to the MVA attenuated strain, the only report performed until now in which the *C12L* gene was deleted from a MVA-BAC suggested that no improvements in the cellular immunogenicity could be made by the deletion of this gene [27].

In this study we have done an in depth characterization of the immunological effects in mice after deleting the IL-18 bp coding gene from the MVA genome. We found that IL-18 bp contributes to immune response evasion during MVA infection, as the deletion enhances T-cell immune responses against vector antigens. Importantly, the deleted vector enhanced the immune response to HIV antigens expressed from recombinant vectors.

## Results

### 1. In vitro characterization of a MVA deleted of the IL-18 bp gene: MVAΔC12L

**Figure 1:**
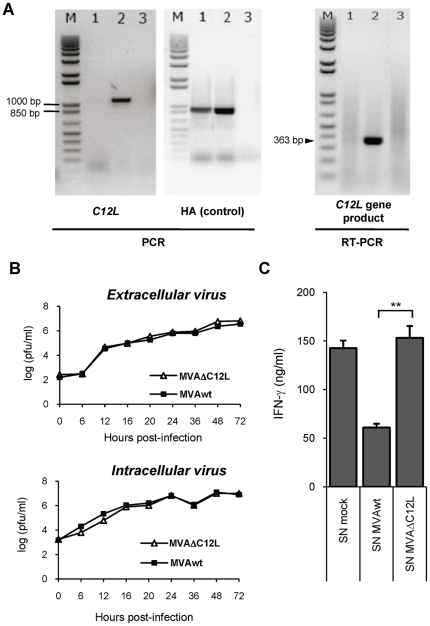
In vitro characterization of MVAΔC12L. (**A**) Corroboration of correct *C12L* gene deletion and abrogation of IL-18 bp expression. DNA and total RNA were extracted from CEFs infected with MVAΔC12L, MVAwt or mock-infected cells (lanes 1, 2 and 3 respectively). *C12L* (left) and *HA* (control gene, middle) sequences were amplified by PCR using specific primers. Absence of *C12L* mRNA (right) was assessed by RT-PCR using specific primers. M: molecular weight marker (1 kb plus DNA ladder, Invitrogen). (**B**) Analysis of virus growth in CEFs after infection at low moi (0.01 pfu/cell), with MVAΔC12L (white triangles) or MVAwt (black squares). Quantification of extra (upper panel) and intracellular (lower panel) virus yields at the different indicated time points was performed as described in Materials and Methods. (**C**) Inhibition of mouse IL-18 biological activity. To evaluate the inhibition of the IL-18-induced IFN-γ production, mice naïve splenocytes were treated with Con A (200 ng/ml) and rIL-18 (5 ng/ml) in the presence of supernatants (SN) from 10^5^ CEFs that had been mock-infected or infected with the indicated viruses. The level of IFN-γ in the culture SN was determined by a standard ELISA assay 24 hs later. Statistically significant differences between MVAwt and MVAΔC12L: **p<0.01.

To analyze the possible role of the *C12L* gene, codifying for IL-18 bp, during MVA infection, we constructed an MVA with a deletion in the *C12L* gene, following the methodology described under Materials and Methods. To verify removal of the *C12L* viral gene we performed a PCR with DNA extracted from CEFs infected with parental or mutant virus (MVAwt or MVAΔC12L), using oligonucleotide primers specific for MVA genomic sequences adjacent to the IL-18 bp gene locus. Figure 1A (left panel), shows an amplified band of nearly 1100 bp from the wild type template, whereas this band was absent in the PCR corresponding to MVAΔC12L. As an internal control, we performed a PCR amplification of another viral gene, the hemagglutinin gene (HA), that produced a band of nearly 900 bp which was present in both DNA templates. To directly verify the absence of *C12L* gene expression, RT-PCR with RNA extracted from CEFs infected with MVAwt or MVAΔC1L was performed. In the right panel of Fig. 1A, a 363 bp fragment specific for the IL-18 bp RNA was only present in the sample from CEFs infected with MVAwt. Previous reports demonstrated that the *C12L* gene was not essential for *in vitro* replication of VACV employing the WR strain [18]. But, as differences in both viral genetic background and in the generation process of the deleted mutant may affect the final virus obtained, we therefore considered important to evaluate the *in vitro* replication capacity of the generated MVAΔC12L mutant. In agreement with the previous report, the virus yields for both intracellular and extracellular virus measured in CEF cells were indistinguishable between parental and mutant virus (Fig. 1B). Previous studies have shown IL-18 binding activity for different Vaccinia strains [17] including MVA, and that MVA expresses a soluble factor that inhibits the IL-12-induced production of IFN-γ by mouse splenocytes [18], suggesting in an indirect form an IL-18 bp activity. Thus, our following aim was to evaluate the loss of function of IL-18 bp in the mutant virus demonstrating that MVA *C12L* gene encodes a protein with a biological activity directly correlated with IL-18. For this, a functional assay was conducted using supernatants of CEFs infected cells to analyze the ability of the C12L protein to inhibit the biological activity of mouse IL-18 (see Materials and Methods). In this assay mouse recombinant IL-18 (rIL-18) was added to mouse splenocytes in the presence of supernatants from MVA infected CEFs and 24 hs later the levels of IFN-γ secreted in the supernatants of the splenocyte cultures were measured by ELISA. Figure 1C shows that preincubation of rIL-18 with supernatants from CEF infected with parental MVA triggered significant reduction of IL-18 biological activity, indicated by reduction in the induction of IFN-γ by mouse splenocytes. The loss of function of this activity in MVAΔC12L was demonstrated by the fact that if rIL-18 was incubated with supernatants from CEFs infected with mutant MVAΔC12L, the inhibition observed was abolished (Fig. 1C).

These findings revealed that we have successfully generated an MVA deletion mutant of *C12L*, that the mutant maintained its replicative capacity in cultured cells compared to the parental virus and we proved that MVA encodes for a protein with a clear biological activity that inhibits the action of IL-18 and this activity is lost by deleting the viral gene.

### 2. MVAΔC12L shows an improved immunogenicity associated with higher magnitude of IFN-γ, IL-2 and cytotoxic specific-CD8^+^ T-cells

**Figure 2:**
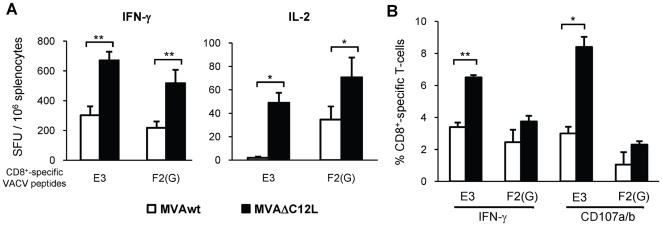
Immunogenicity of MVAΔC12L in BALB/c mice. Groups of four BALB/c mice were i.p immunized with 5×10^7^ pfu of MVAwt (white bars) or MVAΔC12L (black bars), and seven dpi specific-CD8^+^ T-cell responses against the E3 and F2(G) VACV peptides were evaluated in the spleen. (**A**) The magnitude of the specific responses was measured by IFN-γ (left) and IL-2 (right) Elispot assays. Background (RPMI negative control) subtracted results are depicted as mean spot forming units (SFU) per 10^6^ splenocytes ± SD. (**B**) Quality of the response analyzed by ICS. Degranulation of specific-CD8^+^ T-cells was assessed with CD107a/b mAb (cytotoxicity marker) simultaneously with IFN-γ production, after 5 hr of stimulation with VACV peptides Results are expressed as mean % CD8^+^ T-cells ± SD. Statistically significant differences: *p<0.05, **p<0.01.

After the corroboration of the depletion of the IL-18 bp activity from the MVA mutant generated, our next aim was to analyze the modulation of IL-18 bp during the final adaptive immune response generated against the viral vector antigens. To achieve this aim, we firstly analyzed the modulation effects induced after mice inoculation with a high dose of the virus (5×10^7^ pfu) by intraperitoneal (i.p) route. The specific cellular immune response was analyzed 7 days after inoculation, during the acute phase of the response. Figure 2 describes the results found in BALB/c mice (H-2^d^). The specific anti-vector immunogenicity was evaluated against the Vaccinia E3 and F2(G) CD8^+^ T-cell epitopes previously defined. Both epitopes are located on proteins that are expressed early during virus infection [28], similarly to the IL-18 bp product which is expressed before viral DNA replication. Therefore, if the inhibition of the host IL-18 effect mediated by the viral IL-18 bp is causing a depression of the final anti-viral cellular immune response this would be expected to be reflected in the response against these epitopes. Figure 2A (left panel) describes the specific cellular immune response (IFN-γ secreting cells) against the E3 and F2(G) peptides found in the spleen of mice from both groups, where it is shown that for those MVAΔC12L inoculated, significant increments (p<0,01) (2 and 2,5 fold against E3 and F2(G) peptides respectively) were found. Even more, a significant increment was also observed when the IL-2 response was analyzed (Fig. 2A right panel). Thus, after the results obtained by Elispot we did a more in depth analysis by flow cytometry, restimulating the cells for 5 hours with the specific stimulus. We corroborated the results found by Elispot for IFN-γ, but most importantly we could also determine that the immunization with MVAΔC12L also generated an increment in the cytotoxic activity (degranulation evaluated by positive CD107a/b staining), which resulted significantly different for the E3 peptide (p<0,05) (Fig. 2B, right panel).

Next, we analyzed the impact of the deletion of this gene (IL-18 bp) in a different genetic background employing C57BL/6 mice (H-2^b^), which were i.p inoculated with 5×10^7^ pfu/mouse of MVAwt or MVAΔC12L. We selected three CD4 (E9L, H3L and L4R) and one CD8 (B8R) previously defined T-cell epitopes. We selected three CD4 (E9L, H3L and L4R) and one CD8 (B8R) T-cell epitopes previously defined in this mouse model [28], [29].

**Figure 3:**
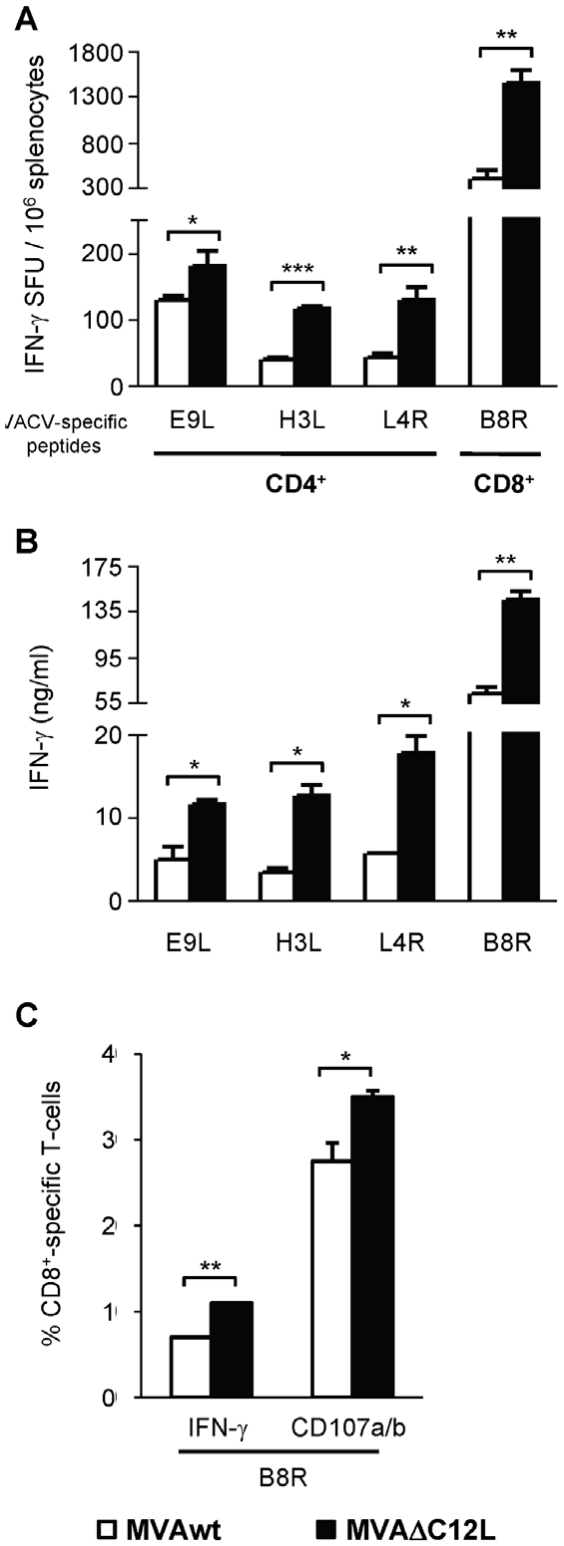
Immunogenicity of MVAΔC12L in C57BL/6 mice. Groups of four C57BL/6 mice were i.p vaccinated with 5×10^7^ pfu of MVAwt (white bars) or MVAΔC12L (black bars) and seven dpi T-cell responses against VACV peptides B8R (CD8^+^-specific), E9L, H3L and L4R (CD4^+^-specific) were evaluated in the spleen. (**A**) The magnitude of the response was measured by IFN-γ Elispot assay after 24 hr stimulation. Background (RPMI negative control) subtracted results are depicted as mean IFN-γ spot forming units (SFU) per 10^6^ splenocytes ± SD. (**B**) IFN-γ production in splenocyte-culture supernatants was evaluated by ELISA after 72 hr stimulation. (**C**) Quality of the response was analyzed by ICS. Degranulation of specific-CD8^+^ T-cells was assessed with CD107a/b mAb together with IFN-γ production, after 5 hr stimulation with the indicated peptides. Results are expressed as mean % of specific CD8^+^ T-cells ± SD. Statistically significant differences: *p<0.05, **p<0.01, ***p<0.001.

The Elispot analysis showed that in the case of the CD8^+^ epitope, the MVAΔC12L generated an increment of 3 to 4-fold (depending of the experiment) (Fig. 3A). It must be noted that the improved immune response, afforded by the mutant MVA in this mouse strain, was enhanced compared to the CD8^+^ T-cell responses studied in BALB/c. In relation to the CD4^+^ T-cell responses after MVAwt inoculation, the highest magnitude was detected against the E9L peptide (expressed early during viral replication) whereas minor responses were detected against the other two epitopes which were expressed at later times. Increments afforded by the MVA mutant against the CD4^+^ peptides (Fig. 3A) were of nearly two-fold in the IFN-γ secreting cells determined by Elispot after a restimulation period of 24 h, whereas extending this period to 72 h and quantifying levels of specific IFN-γ secreted, the differences between both vectors were pronounced (Fig. 3B). Thus, increments of 2.5 (H3L) to 3 fold (L4R) in relation to MVAwt induced responses were observed. Moreover, in this model we also evaluated if MVAΔC12L modulated the cytotoxicity of the CD8^+^ specific response, with the findings of a significant increase in B8R specific CD107a/b cells in those mice inoculated with the mutant MVA (Fig. 3C).

### 3. MVAΔC12L elicits higher cellular responses at lower doses of immunization and by different immunization routes

**Figure 4:**
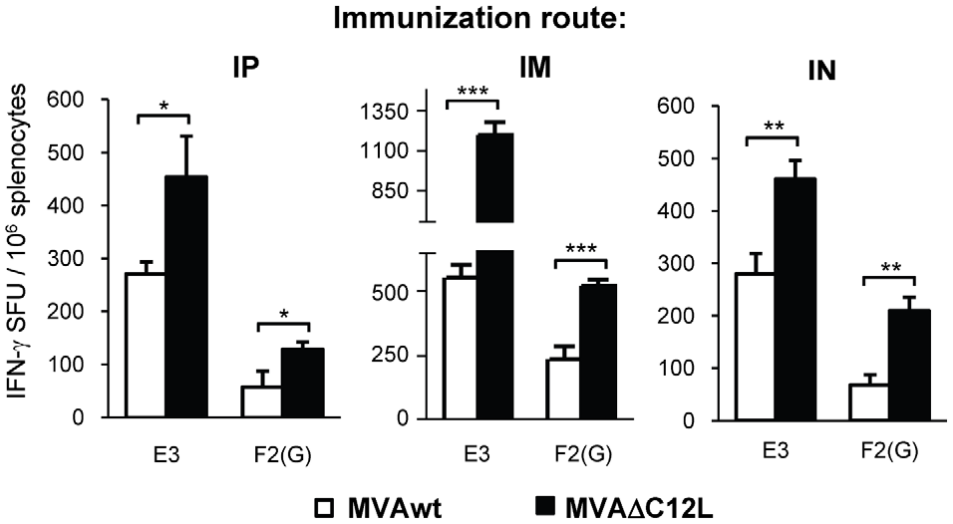
MVAΔC12L elicits higher cellular responses at lower doses of immunization and by different immunization routes. Groups of four BALB/c mice were immunized by intraperitoneal (IP) intramuscular (IM) or intranasal (IN) route with 10^7^ pfu of MVAwt (white bars) or MVAΔC12L (black bars) and seven dpi specific-CD8^+^ T-cell responses against E3 and F2(G) peptides were evaluated in the spleen. The magnitude of the response was measured by IFN-γ Elispot assay after 24 hr stimulation. Background (RPMI control) subtracted results are depicted as mean IFN-γ spot forming units (SFU) per 10^6^ splenocytes ± SD. Statistically significant differences: *p<0.05, **p<0.01, ***p<0.001.

The results of the experiments described above clearly showed that the deletion of the IL-18 bp codifying gene, produced beneficial effects on the immunogenicity generated by MVA. Those experiments were performed by inoculating mice with 5×10^7^ pfu, a somehow high viral dose, compared with the standard doses (10^7^ pfu) employed in the majority of the MVA studies performed in mice and by i.p route. Thus, our following aim was to analyze if at lower doses of immunization and after application of the vector by other routes, the deletion of the IL-18 bp still had an improved effect on the MVA vaccine potential. In these experiments a five-fold lower viral dose was applied to BALB/c mice (10^7^ pfu/mouse) by alternative routes, such as the intramuscular (i.m) and the intranasal (i.n) and, for comparison, we also evaluated the responses generated after this lower viral dose by the i.p route. In the left panel of Figure 4 the specific response (number of IFN-γ secreting cells) detected against both CD8^+^ peptides (E3 and F2(G)) were significantly incremented in the MVAΔC12L i.p inoculated mice. Of note, the magnitude found was comparable to that recorded after the 5×10^7^ pfu dose (see Fig. 2A) specially for the E3 peptide (immunodominant), whereas for F2(G) (less immunogenic in this model) lower responses were detected. Notably, the i.m route resulted the most effective in relation to the magnitudes generated, strengthening the response in comparison to the i.p route (1200 vs 450 SFU/10^6^ for MVAΔC12L and 500 vs 250 SFU/10^6^ for the MVAwt). Importantly, we could also find an improvement in the response with the mutated virus after the i.n immunization (Fig. 4 right panel), a route with high relevance to the induction of mucosal immune responses after MVA immunizations [30], [31], [32].

Thus, the findings shown in Figure 4 demonstrated that the enhancements in the cellular immune responses generated by MVAΔC12L were also exerted after the inoculation of lower viral doses and by different immunization routes.

### 4. Analysis of the immune response generated by MVAΔC12L in local draining lymph nodes to the site of immunization

**Figure 5:**
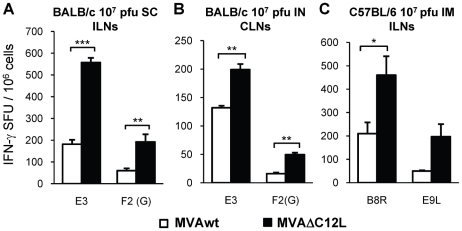
Analysis of the immune response generated in local draining lymph nodes (LNs) to the site of immunization. Groups of four mice were immunized as indicated in the bar charts with 10^7^ pfu of MVAwt (white bars) or MVAΔC12L (black bars) and seven dpi specific T-cell responses against the indicated peptides were evaluated in the regional draining LNs to the different immunization routes as depicted in the Figure. The magnitude of the response was measured by IFN-γ Elispot assay after 24 hr stimulation. Background (RPMI control) subtracted data are depicted as mean IFN-γ spot forming units (SFU) per 10^6^ cells ± SD. SC: subcutaneous; IN: intranasal; IM: intramuscular; ILN: inguinal lymph nodes; CLN: cervical lymph nodes. Statistically significant differences: *p<0.05, **p<0.01, ***p<0.001.

The primary adaptive immune response to most pathogens and vaccines is initiated in regional lymph nodes draining peripheral sites of antigen exposure. Lymph nodes are highly organized structures designed to efficiently transfer antigen transported from the periphery to node-resident cells specialized in acquiring, processing and presenting antigen to lymphocytes. It is uncertain how antiviral lymphocytes are activated in draining lymph nodes, the site where adaptive immune responses are initiated. Recent studies have demonstrated that naïve CD8^+^ T-cells rapidly migrated to VACV-infected cells in the peripheral interfollicular region and then formed tight interactions with dendritic cells (DCs), leading to complete T-cell activation [33]. It was also shown how the administration route can target different APCs that differentially shape the virus-specific cell-mediated immune response of both CD4^+^ and CD8^+^ cells, in such a way that the accessibility of MVA antigens to different APCs at the site of immunization dictates the occurrence and extent of cellular immunity [34]. Thus, considering the importance that the draining lymph nodes (LNs) to the site of inoculation have in the final outcome of the immune response, our following goal was to analyze MVA-specific T-cell responses in the regional draining LNs after different immunization routes. Responses were evaluated in the inguinal LNs, (ILNs) after sub-cutaneous (s.c), and i.m inoculations and cervical LNs (CLNs) after i.n immunizations. For the three routes evaluated, we continued to observe differences between both MVAs, finding improved T-cell responses in those groups MVAΔC12L immunized. Importantly, we found that after s.c inoculation in BALB/c differences between both MVAs were incremented in the ILNs, resulting in a three-fold superior improvement of the responses against E3 and F2(G) peptides (Fig. 5A). Therefore, in line with previous reports we found that the final T-cell response was influenced by the APCs at the site of immunization, as differences between both vectors detected in the spleen, after administration by the i.p route with the same or even higher viral doses (1 to 5×10^7^ pfu/mouse), were always nearly 2-fold. After i.n immunization, increments in the responses found in the CLNs of MVAΔC12L were nearly 2-fold (Fig. 5B), following a similar pattern to that previously detected in the spleen for this mouse strain. In C57BL/6 mice we could also find in the draining ILNs an incremented response against both peptides analyzed (B8R and E9L) when MVAΔC12L was applied (Fig. 5C).

The findings shown in Figure 5 revealed that the CIR enhancements generated by MVAΔC12L in infected mice were also detected in the draining lymph nodes to the site of inoculation of the viral vector.

### 5. MVAΔC12L improves T-cell memory responses conferring a higher grade of protection than parental MVA after challenge with virulent VACV (WR)

Memory CD8^+^ T-cells are an important component of acquired immunity to viruses and other pathogens and represent the main final aim of T-cell vaccines. Their principal characteristic is the capacity to persist for extended periods, responding more rapidly and more vigorously than naïve T-cells when they reencounter the same antigen [35], [36]. Thus, we proceeded to define if after contraction of the specific immune response triggered by both vectors, differences in memory responses induced by MVAΔC12L versus MVA still persist with time.

**Figure 6:**
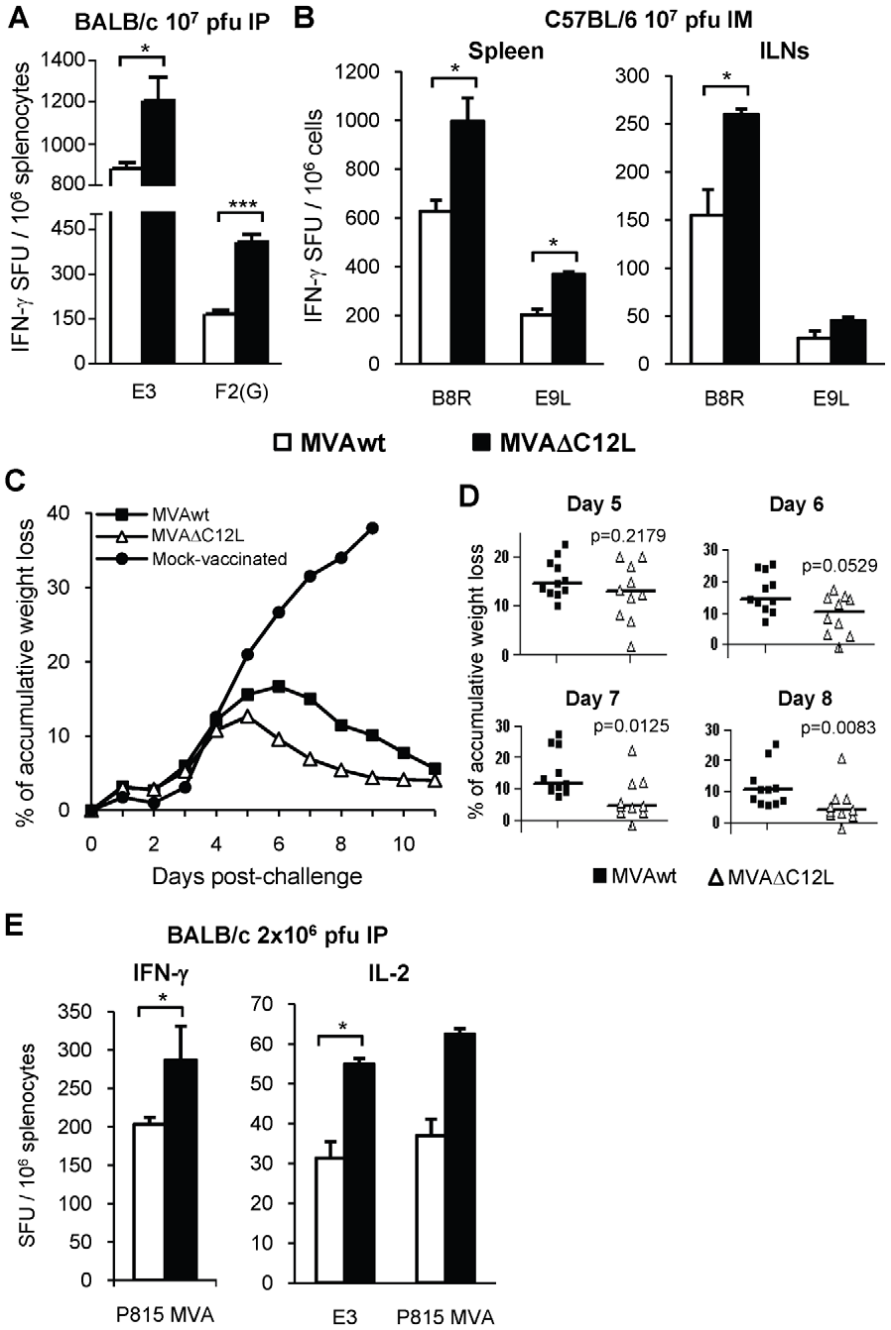
MVAΔC12L improves T-cell memory responses conferring a higher grade of protection against a VACV challenge. (**A**) Groups of four BALB/c mice were i.p immunized with 10^7^ pfu of MVAwt or MVAΔC12L. At 40 dpi the magnitude of the T-cell response in the spleen was measured by a more sensitive IFN-γ Elispot assay as described in Materials and Methods. (**B**) Groups of four C57BL/6 mice were i.m inoculated with 10^7^ pfu of the indicated vectors and at 40 dpi the magnitude of the response was evaluated by IFN-γ Elispot assay against the CD8^+^ (B8R) and CD4^+^ (E9L) VACV peptides in the spleen and ILNs. (**C**) Groups of 11 BALB/c mice were i.p vaccinated with 10^6^ pfu of MVAwt, MVAΔC12L or mock immunized and 45 dpi all animals were intranasally challenged with 2×10^6^ pfu of the VACV WR strain. Mice were daily weighed during 12 days and the mean % of accumulative weight loss for each group was calculated (see Materials and Methods). (**D**) Detailed % of accumulative weight loss of individual animals within each indicated group at 5, 6, 7 and 8 days post-challenge, median values are shown. (**E**) Groups of 4 BALB/c mice were i.p immunized with 2×10^6^ pfu of the vectors and 40 dpi the response against E3 peptide and/or P815-MVA infected cells was evaluated by IFN-γ and IL-2 Elispot assays. Background (RPMI control) subtracted results are depicted as mean spot forming units (SFU) per 10^6^ cells ± SD. Statistically significant differences: *p<0.05, ***p<0.001.

Figure 6 (A–B) shows the specific immune responses detected 40 days after immunization with a dose of 10^7^ pfu/mouse. It must be noted that, for these experiments, the Elispot assays were performed with a kit of higher sensitivity than in previous figures (detecting responses with a sensitivity two to three fold higher), in order to detect with a high precision the memory responses that are of lower magnitude with respect to those of the acute phase. Importantly, after 40 days, improvements in the immunogenicity induced by MVAΔC12L were still detected in both mouse strains. Significant differences in immune responses induced by both vectors were found for VACV-CD8^+^ peptides in BALB/c and C57BL/6 mice, and in this last strain differences against the E9L CD4^+^ peptide were also detected. In the C57BL/6 mice we also analyzed immunity detected in the ILNs, finding a similar trend in the responses generated by both MVA vectors. As observed in Fig. 6 A–B, during the memory phase, MVAΔC12L produced T-cell responses nearly two-fold superior (increments varied from 1,4 to 3 fold) than those induced after MVAwt inoculation.

Then, our next aim was to evaluate if the deletion of IL-18 bp could improve the MVA vaccine potential, monitoring whether the different levels of memory T-cell responses were correlated with an enhanced protection against a virulent strain of VACV. For this, three groups of 10–11 BALB/c mice per group were firstly i.p immunized with 10^6^ pfu/mouse of MVAΔC12L, MVAwt or mock-treated, and 45 days later they were challenged by i.n inoculation with 2×10^6^ pfu of Vaccinia virus WR (this is about 20-fold the LD50). In these experiments, a low dose of MVA immunization was used to reduce the levels of neutralizing antibodies to the virus, in order to perform a more direct correlation between the cellular immunity generated and the protection afforded.

In mock-vaccinated control mice VACV infection resulted in the onset of respiratory disease, high grade of weight loss, detecting >30% of accumulative weight loss at day nine 9 post-infection (p.i) (Fig. 6C). As it must be expected, mice previously immunized with MVA vectors showed a clear reduction in their illness signs, compared to mock-vaccinated ones. Significant differences in illness signs between MVA inoculated groups and mock animals were detected from day 6 to day 8 and at day 9 mock mice had to be sacrificed (Fig. S1).

MVAΔC12L group reached their maximum weight loss at the fifth day (13%) and then began to recover rapidly; while in the MVAwt group the loss of weight continued until the sixth day inclusive (17%) (Fig.6C). It must also be noted, that in these mice the recovery was more slowly from day 5 to day 8 p.i, with significant differences between both groups at days 7 and 8 p.i (Fig. 6D). Moreover, significant differences in illness signs between both groups were also found at days 7 and 8 p.i (Fig. S1). To provide evidence that prior to the time of challenge the MVAΔC12L infected mice generated an enhanced cellular immunity over the MVA group, splenocytes from BALB/c mice i.p inoculated with 2×10^6^ pfu of the viruses, were isolated and tested for cellular responses against VACV antigens (P815 cells infected with MVA and E3 peptide). Importantly, significant higher numbers of both IFN-γ and IL-2 secreting cells were detected in the MVAΔC12L group (Fig. 6E).

These data showed that the improvements in the cellular immune responses generated after MVAΔC12L immunization were maintained for longer times, during the memory phase, and moreover this enhancement in the immune response was reflected in a major protective capacity against an intranasal WR challenge.

### 6. Quality of the specific anti-VACV response induced in the absence of IL-18 bp viral activity

**Figure 7:**
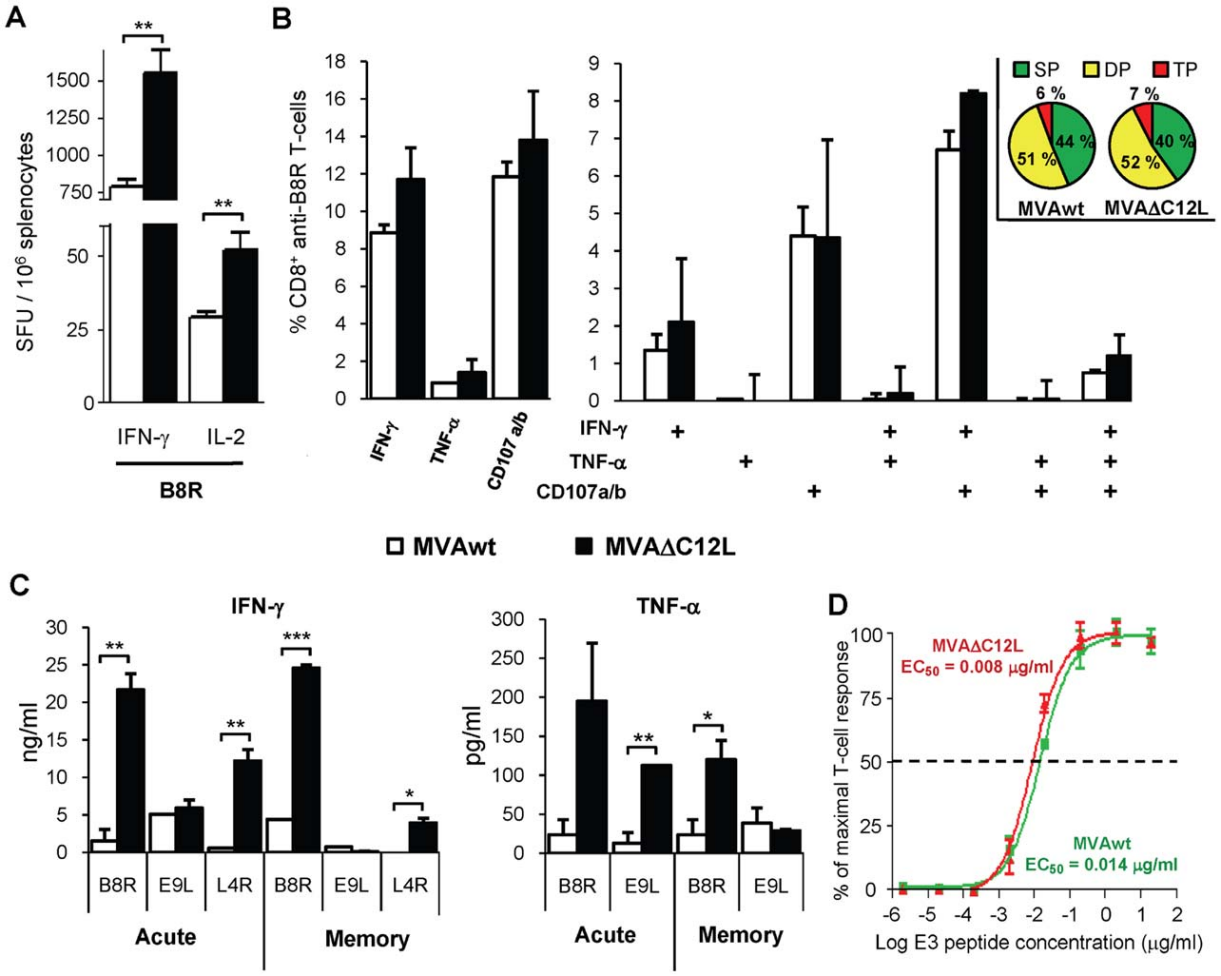
Quality characterization of the specific T-cell anti-VACV immune response. (**A**) Groups of four C57BL/6 mice were i.m immunized with 10^7^ pfu of MVAwt (white bars) or MVAΔC12L (black bars), and seven dpi the magnitude of the T-cell response against B8R peptide in the spleen was measured by IFN-γ and IL-2 Elispot assays and (**B**) degranulation of specific-CD8^+^ T-cells was assessed with CD107a/b staining simultaneously with IFN-γ and TNF-α production by ICS, after 5 hr of stimulation with B8R peptide. Analysis of the total B8R specific-CD8^+^ T-cells expressing each of the functions (left panel) and polyfunctionality of the specific-CD8^+^ T-cells were performed (right panel). Bars represent the frequency of CD8^+^ T-cells expressing the particular combination of functions indicated on the *x* axis. Results are expressed as mean % CD8^+^ anti-B8R T-cells ± SD. Each pie chart represents the mean contribution of each effector function to the total response. Green sectors stand for CD8^+^ cells positive for only one function (SP), yellow sectors stand for bifunctional CD8^+^ cells (DP), and red sectors stand for trifunctional cells (TP). (**C**) Groups of four C57BL/6 mice were i.m immunized with 10^7^ pfu of MVAwt or MVAΔC12L and 7 (acute) or 35 (memory) dpi, specific cytokine production in splenocyte-culture supernatants was evaluated by ELISA after 72 hr stimulation with the indicated peptides. (**D**) Splenocytes from BALB/c mice i.m immunized with 10^7^ pfu of MVAwt (green) or MVAΔC12L (red) were assayed by IFN-γ Elispot against serial dilutions of VACV E3 peptide. T-cell functional avidity is defined as the concentration required to achieve half-maximal of the response (EC_50_). Data represents the percentage of the maximal response (net number of SFU per 10^6^ cells stimulated with a peptide concentration of 20 µg/ml). Statistically significant differences: *p<0.05, **p<0.01, ***p<0.001.

Then, our following aim was to do a more in depth analysis to characterize the quality of the cellular immune responses generated by the MVAΔC12L mutant. Based on the results described in the previous sections, the i.m immunization route was selected and the viral dose employed was 10^7^ pfu/mouse. Figure 7A depicts the results from Elispot assays evaluating the number of B8R specific T-cells that secrete IFN-γ or IL-2, demonstrating significant increments in both CD8^+^T-cell types in those C57BL/6 mice immunized with MVAΔC12L. Figure 7B shows data of ICS analysis of the different functions exerted by specific B8R CD8^+^ T-cells found in the spleen of MVAwt or MVAΔC12L immunized mice. When total IFN-γ, TNF-α, or CD107a/b positive cells against the B8R peptide were analyzed (Fig. 7B left panel) it can be seen that MVAΔC12L generated an increment in the proportion of cells positive for any of the functions. These results were in concordance with data shown in Figure 3C; although in that experiment the i.p route was used and the viral dose was five times higher. The polyfunctionality pattern of the T-cell response indicated that the MVAΔC12L immunization did not change in relation to that generated after MVAwt immunization, as similar percentages of cells positive for the three, two or one of the functions studied were found in both groups of mice (Fig. 7B right panel).

We also analyzed the capacity of the specific T-cells to secrete IFN-γ and TNF-α in both acute (7 days p.i) and memory phases (40 days p.i) of the response (Fig. 7C). Data showed that at both the peak of the T-cell response and also at late times, when the contraction of the response had already occurred, CD8^+^ T-cells (anti-B8R) from MVAΔC12L inoculated mice produced higher levels of IFN-γ and TNF-α than MVA mice. In relation to the CD4 peptides (E9L and L4R), increased levels of both cytokines were found for the mutant MVAΔC12L restimulated splenocytes during the acute phase, whereas at the memory phase as the intensity of the CD4^+^ T-cell response was diminished we could only detect differences in IFN-γ levels. Finally, we decided to analyze the T-cell functional avidity as another important T-cell function directly correlated with its quality. This T-cell capacity is a reflection of the efficiency of the effector cells, as it measures the ability of the cells to recognize its specific antigen at low concentrations. Thus, to investigate if the MVAΔC12L immunization could have altered the avidity of the T-cell response, we evaluated the functional avidity of CD8^+^ E3-specific T-cells after i.m immunization of BALB/c mice with 10^7^ pfu of each MVA vector. For this, Elispot assays at different peptide concentrations were performed. Fig. 7D shows that the curves obtained with splenocytes from both mice groups (MVAwt or MVAΔC12L immunized) were similar, showing no differences among the functional activities. Thus, values for 50% of the maximal T-cell responses (EC_50_) did not differ significantly among groups: EC_50_ values calculated with a sigmoid dose-response curve (GraphPad software) were 0,014 (MVAwt) and 0,008 µg/ml (MVAΔC12L).

These findings corroborated the results shown in previous sections demonstrating that after MVAΔC12L immunization a higher proportion of anti-viral specific T-cells secreting IFN-γ, TNF-α, IL-2 or with cytotoxic capacity were generated. Moreover, we demonstrated that this enhancement was not at the expense of the quality of the response, analyzed by polyfunctional or avidity properties.

### 7. MVAΔC12L induced higher cellular responses against recombinant HIV antigens compared to MVA in DNA prime/MVA boost immunizations

The renewed interest for the development of poxvirus-based HIV vaccines has been boosted by the recent results obtained from the phase III RV-144 trial in Thailand [37]. While these studies showed modest efficacy (31%), they provide for the first time evidence of a candidate HIV vaccine capable of preventing HIV infection. Since a poxvirus vector (canarypox) was used in the RV-144 trial and the T-cell immune response was poor, it suggested that a poxvirus vector with more potent capacity to induce T-cell responses to HIV antigens might be more effective. Thus, the last aim of this work was to analyze if the enhancement observed against VACV antigens after deletion of IL-18 bp gene could also be induced against different HIV recombinant genes expressed from MVAΔC12L vectors.

**Figure 8:**
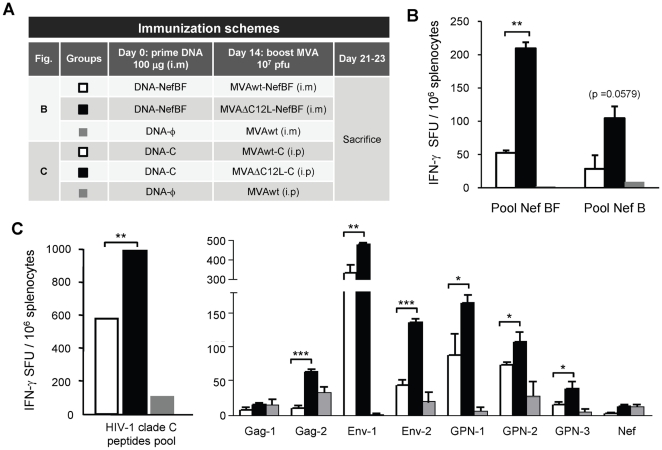
MVAΔC12L induced strengthened cellular responses against recombinant HIV antigens compared to MVA after DNA/MVA immunizations. (**A**) Description of the different vectors employed in the DNA/MVA immunization schemes applied, each group consisted of three to four BALB/c mice. (**B**) Seven days after the boost, specific-CD8^+^ T-cell responses against NefBF (homologous) or NefB (heterologous) peptides were evaluated in the spleen. (**C**) Ten days after the boost specific responses against HIV-1 subtype C antigens were evaluated in the spleen. To the left it is depicted the responses found against the total pool of peptides analyzed, in the right panel it can be seen the detailed analysis showing the pool of peptides targeted comprising the different HIV antigens that are expressed from the vectors. The magnitude of the response was measured by IFN-γ Elispot assay after 24 hr stimulation. Background (RPMI control) subtracted results are depicted as mean IFN-γ spot forming units (SFU) per 10^6^ splenocytes ± SD. Statistically significant differences: *p<0.05, **p<0.01, ***p<0.001.

For these experiments, immunizations based on DNA prime/MVA boost schemes were applied, but now employing recombinant MVA vectors expressing NefBF [38] or a recombinant MVA expressing codon-optimized Env as a monomeric gp120 and a polyprotein Gag-Pol-Nef from clade C (referred as MVA-C) [39]. The immunization schemes are described in Figure 8A. Fig. 8B–C shows the HIV specific responses detected 7–10 days after the last immunization. When the NefBF antigen was used, it can be clearly seen that if the booster dose was MVAΔC12L-NefBF, the reactivity against NefBF peptides (overlapping peptides representing the entire protein) was significantly improved (Fig. 8B), and even more, a higher -albeit not significant- level of cross-reactivity against B peptides was also found in mice that received the DNA-NefBF/MVAΔC12L-NefBF scheme.

When the recombinant MVA-C vectors were administered at booster, IL-18 bp modulation on the immunity against a wider range of HIV antigens could be observed. As shown in Fig. 8C the magnitude of the T-cell responses, measured against the total pool of peptides represented in the HIV antigens, was higher in splenocytes from mice boosted with MVAΔC12L than boosted with MVA. The right panel depicts the responses against the different HIV peptide-pools contained within the four different HIV proteins. Remarkably, the enhancement produced by MVAΔC12L-C was observed for most of the peptide-pools evaluated. Of note, significant higher responses against five out of eight pools were found in the MVAΔC12L-C group (Fig. 8C).

These findings (Figure 8) show that optimization of the immune response generated after MVAΔC12L can also be achieved against HIV proteins from different subtypes, generating HIV responses with higher magnitude and amplitude after a DNA prime/MVA boost scheme. These data are of significance in the design of future poxvirus-based HIV vaccines.

## Discussion

There is a substantial need to improve the immunogenicity of MVA-based vaccines, which are currently being developed for use against a number of prominent infectious diseases including AIDS, malaria, and tuberculosis, as well as human cancers [40].

The proof of concept that MVA vaccine potential can be improved after removing viral genes involved in host immune response evasion was demonstrated in previous studies in which genes as the one encoding interleukin 1β (IL-1β)-binding protein, or the *A41L* and *C6L* genes which blocks the action of CC-chemokines and IFN-β were removed [14], [15], [41]. In the present study we demonstrate for the first time that a MVA deleted of the gene coding for the IL-18 bp (*C12L*) showed an enhanced T-cell immunogenicity against both CD8^+^ and CD4^+^ T-cell VACV peptides, and more importantly this optimization was also exerted against HIV recombinant antigens.

It was previously demonstrated that IL-18 bp was produced in response to VACV (WR strain) infection *in vitro* [17], [18]. The relevance of the *C12L* gene during infection of mice with this viral strain, was shown by an augmentation of NK cytotoxicity and CTL responses after infection with a *C12L* VACV deletion mutant [25]. And more recently, it has been demonstrated that deletion of the viral IL-18 bp lessened the virulence of the Tiantan VACV strain in both mice and rabbit models [26]. It was previously reported that the MVA genome encoded an IL-18-binding activity [17]. However, here we described for the first time that MVA encodes for a protein with a clear biological activity that inhibits the action of IL-18, and that deletion of the *C12L* viral gene (*008L* in MVA) abolished this inhibitory activity. Then, the first experiments performed in BALB/c mice (Fig. 2) indicated the importance of IL-18 modulation on MVA immunogenicity. Thus, mice infected with MVAΔC12L, and therefore in the absence of an inhibitory effect against host IL-18, generated responses against CD8^+^ epitopes of a higher magnitude, rendering two-fold increments in the number of specific IFN-γ and IL-2 secreting cells against the E3 and F2(G) VACV peptides.

In C57BL/6 mice, these observations were corroborated, finding significant T-cell enhancements that reached three to four-fold increments against the immunodominant CD8^+^ B8R peptide, and also a positive modulation against CD4^+^ epitopes. A critical function of the CD8^+^ T-cells is their cytotoxic capacity, a parameter which directly correlates with protective anti-viral immunity. Importantly, we found that in both mouse strains BALB/c and C57BL/6, MVAΔC12L administration also improved the number of CD8^+^ T-cells with cytotoxic properties (cells positive for the CD107a/b markers). The only previous data indicating a direct evidence of an augmentation of the CTL activity after deletion of the *C12L* gene, was documented for the WR strain [25].

In a relative recent publication in which the *C12L* gene was deleted from the MVA genome employing the methodology of recombination-mediated genetic engineering of a bacterial artificial chromosome (BAC), the authors did not find an improvement in the CD8^+^ T-cell immunogenicity [27]. However, in that study a single viral dose and administration route were analyzed (10^6^ pfu by intradermal (i.d) route), in contrast with the different routes and varied viral doses that we have analyzed in the present study. It must also be noted that, after the application of the BAC technology, among the five VACV deleted genes already described in previous works [14], [15], [18], [42], [43], only the deletion of the *B15R* gene was associated with an improvement in the MVA immunogenicity.

The efficacy of MVA immunization has been investigated in several animal models and by different immunization routes [32], [40], [44], [45]. In relation with this, the relevance that the application of distinct routes of immunization could have on the final adaptive cellular response induced after MVA immunization was analyzed in a recent study [34]. It was found that MVA administration after i.d. or i.m routes target different APCs that differentially shape the virus-specific cell-mediated immune response. In the present study, the improved immunogenicity described for the MVAΔC12L mutant vector was corroborated after the inoculation of different viral doses (from 5×10^7^ to 2×10^6^ pfu/mouse) and even more, this optimization was verified after i.p, i.m or i.n immunizations. In relation to the impact that the inoculation route could have on the final adaptive immune response generated, comparing the i.p vs the i.m routes, we found that after this last route a significant enhancement (of nearly 3 times) on the final magnitude of the specific responses detected in the spleen were observed against both peptides and in animals inoculated with MVA or MVAΔC12L. A possible explanation to the results obtained here may be differences in the principal types of APCs that are participating in the initiation of the immune response after i.p or i.m inoculation. Another factor that may be influencing the differences observed between the i.p and i.m routes, may be a differential pattern of the MVA viral gene expression. Therefore, previous studies have demonstrated higher levels of gene expression post-intramuscular inoculation than those recorded after i.p inoculation [46].

Given the application of MVA as a vaccine vector, the observation that the beneficial immunogenicity effects after the deletion of the *C12L* gene were also observed during the memory phase is an issue of high relevance. Our results suggest the importance of IL-18 to induce and longer maintain the improvements induced in the anti-viral T-cell immune responses. Early exposure to distinct cytokines most commonly influences the balance between the development of short-lived, terminally differentiated effector cells and memory precursors CD8^+^ T-cells [47]. Beyond the induction phase, cytokines contribute to the regulation of the contraction of the response, as well as the long-term maintenance of memory CD8^+^ T-cells. It has been described that increasing the amount or duration of IL-12 stimulation of CD8^+^ T-cells results in elevated expression of the transcription factor T-bet (Tbx 21), which enforces an effector (CD127^lo^KLRG-1^hi^) phenotype [48]. On the other hand, signaling by cytokines with a common γ_c_ cytokine receptor: IL-2, IL-7, and IL-15, activate prosurvival signals and up-regulation of the anti-apoptotic molecule, Bcl-2 [49]. Regarding the relevance of IL-18 in the promotion of T-cell memory responses, a recent paper indicated that a positive regulatory loop involving IFN-γ and IL-18 signaling contributes to the accelerated memory CD8^+^ T-cell proliferation during a recall response to antigens presented by DCs [50]. Although another study described that, despite the induction of IL-18-related genes during the contraction phase, they do not play major roles in regulating the dynamics or function of the T-cell response to *Listeria Monocytogenes* or VACV infection [51].

The biological relevance of the immunization with the MVAΔC12L mutant was also evaluated by analyzing its efficacy in conferring protection against a challenge with the virulent VACV WR strain in the well established i.n challenge BALB/c model [52]. In these experiments, mice were inoculated with low immunizing doses (2×10^6^ pfu, by i.p route), after which low levels of anti-VACV antibodies were induced [7], in order to have a window for a more direct correlation between T-cell immunity induced and protection afforded. But, it must be taken it into account that although low levels of anti-VACV antibodies are induced after that viral dose, possibly higher levels may be present in MVAΔC12L inoculated mice, as it was found in mice inoculated with 10^7^ pfu/mice (data not shown). The challenge experiments showed that mice that received MVAΔC12L presented an increased protection against the WR challenge at the memory T-cell phase, highlighting the improved protective capacity of the T-cell responses generated by the IL-18 bp deleted vector. Previous studies performed with other genes deleted MVA mutants also correlated the improvements on the cellular immunity with an enhancement in their protective capacity [14], [15]. To notice, this is the first study in which the *C12L* gene effects on the T-cell memory responses are analyzed, as in other previous works in which the *C12L* gene was characterized, immune responses were only studied during the early phase.

When we analyzed the vaccine potential of the MVAΔC12L with respect to recombinant expressed antigens, in particular HIV antigens, we applied the MVA dose as a booster in relation to the recombinant antigens (in a DNA/MVA scheme). We employed two MVAΔC12L recombinants one expressing a single HIV protein: NefBF [38] and another one expressing a codon-optimized Env as a monomeric gp120 and a *syn* polyprotein Gag-Pol-Nef of HIV-1 from clade C [39]. Importantly, in both cases the delivery of the HIV antigens during the booster dose from the MVAΔC12L vectors generated an enhancement of the specific cellular response, and moreover the breadth of the HIV-responses was improved as positive T-cell responses against a wider spectrum of peptides were detected for both recombinants. For the NefBF antigen we have previously reported that after DNA/MVA immunization a low immunogenicity was detected against NefBF, which could be incremented if 3×DNA sequential immunizations were applied during priming [38]. A fact to be denoted is that when the MVAΔC12L-NefBF was applied at boosting after the DNA priming doses, a significant increment in the response against Nef HIV was achieved, comparable to that found when three DNA priming doses were applied. A possible mechanism explaining why the absence of IL-18 bp viral activity at the moment of the MVA boost can mediate an enhancement of the DNA-primed HIV responses, may be the positive regulatory loop involving IFN-γ and IL-18 signaling recently proposed to be contributing to the accelerated memory CD8^+^ T-cell proliferation during a recall response to antigens presented by DCs [50].

In summary, these results showed that the MVA *008L* (*C12L*) gene encodes for a protein with a clear biological activity that inhibits the action of IL-18, and that the deletion from its genome abolished this inhibitory activity. Analysis of the *in vivo* effects of IL-18 bp after immunization with MVAΔC12L showed that at early times post-inoculation higher numbers of T CD8^+^ and CD4^+^ anti-VACV IFN-γ and IL-2 secreting cells were generated. Importantly, we found that MVAΔC12L administration also improved the number of CD8^+^ T-cells with cytotoxic properties. At later times post-immunization MVA inoculated mice still maintained higher CD8^+^ and CD4^+^ T-cell VACV-specific responses, which were correlated with an increased protection against an i.n WR challenge. Finally, in DNA prime/MVA boost regimes, the delivery of HIV antigens during the booster dose from a MVAΔC12L vector generated an enhancement of the T-cell response against the HIV proteins, improving the breadth, as significant responses against a wider spectrum of antigens were detected. These results are of high relevance for the design of new optimized poxvirus vector based vaccines.

## Materials and Methods

### 1. Cells

Primary cultures of chicken embryo fibroblasts (CEFs) were prepared by the Tissue Culture Section of the INTA-Castelar Virology Institute, from 11 days old specific pathogen free (SPF) embryos (Instituto Rosenbusch, Argentina) and maintained in 199 Earle Medium supplemented with 2.95 mg/mL tryptose phosphate broth (BD, Sparks, MD), 2.2 mg/mL sodium bicarbonate (ICN Biomedicals Inc., Irvine, CA), 0.3 mg/mL L-glutamine, 50 µg/mL gentamicin, 66 µg/mL streptomycin, 100 U/mL penicillin and 10% fetal bovine serum (FBS, Internegocios, Argentina).

Stable cell lines employed in the study were: BSC-40 (epithelial adherent cell-line derived from African green monkey kidney cells, ATCC Cat. N° CRL-2761); BHK-21 (fibroblast adherent cell-line derived from hamster Syrian golden kidney cells, ATCC Cat N° CCL-10) and P815 (DBA-2 mice mastocytoma cells, ATCC Cat N° TIB-64); DF-1 cells (a spontaneously immortalized chicken embryo fibroblast cell line, ATCC, Manassas, VA).

Cells were maintained at 37°C in a 5% CO_2_ atmosphere in Dulbecco's Modified Eagle's Medium (DMEM) supplemented with 10% FBS (DMEM 10% FBS).

### 2. Construction of plasmid transfer vectors

In order to obtain the different MVAΔC12L vectors employed in this study two transfer plasmid vectors were generated. For the construction of the pΔC12L plasmid transfer the flanking regions of *008L* gene (coding for IL-18 bp, nt 13052–13414) were amplified by PCR and cloned sequentially into pBlueScript (Stratagene). The left and right flanks were 330 and 374 bp length encompassing MVA genomic positions 12730–13059 and 13416–13789, respectively. The pairs of primers used were 008i1 GAATTCGTTGTTTACTCAAAACG/008i2 GGATCCGAAGTAGTGCGTGCTAC and 008d1 GCGGCCGCCTTGCCAAAATATCAC/008d2 GAGCTCCTATAATGATTATATAG, for left and right flanks respectively. Recognition sites for restriction enzymes used for cloning are showed underlined. The intermediate plasmid obtained was named pBS-008. Then, the *lac Z* gene (coding the β-galactosidase enzyme [βGAL]) under regulation of poxviral H6 promoter was excised from a plasmid available in our laboratory and it was subcloned into pBS-008 to obtain pΔC12L transference vector. The identity of all plasmids was verified by DNA sequencing using an ABI PRIS® 310 Genetic Analyzer (Applied Biosystems, Japan).

The plasmid transfer vector pGem-RG-C12L, used for the construction of recombinant virus MVAΔC12L-C in which C12L ORF has been replaced for a GFP expression cassette, was obtained by the sequential cloning of four DNA fragments containing dsRed2 and rsGFP genes and C12L recombination flanking sequences into the plasmid pGem-7Zf(−) (Promega). The dsRed2 gene under the control of the synthetic early/late promoter (783 bp) was obtained by digestion of plasmid pG-dsRed2 (encoding dsRed2 and rsGFP genes) with Xba I and Eco RI and inserted into the Xba I/Eco RI-digested pGem-7Zf(−) to generate pGem-Red. The rsGFP gene under the control of the synthetic early/late promoter was amplified by PCR from plasmid pG-dsRed2 with oligonucleotides GFP-C (5′-GTTGGATCGATGAGAAAAATTG-3′) (Cla I site underlined) and GFP-B (5′-CTATAGGATCCTCAAGCTATGC-3′) (Bam HI site underlined) (828 bp), digested with Cla I and Bam HI and inserted into plasmid pGem-Red previously digested with Cla I and Bam HI to obtain pGem-Red-GFP. MVA genome was used as the template to amplify the left flank of *C12L* gene (375 bp) with oligonucleotides fiC12L-BF (5′-AGGATGGATCCCTTGCCAAAATAT-3′) (Bam HI site underlined) and fiC12L-BR (5′-TATATGGATCCTTGCAATTAAG-3′) (Bam HI site underlined). This left flank was digested with Bam HI and cloned into plasmid pGem-Red-GFP previously digested with the same restriction enzyme to generate pGem-RG-fiC12L. The right flank of *C12L* gene (404 bp) was amplified by PCR from MVA genome with oligonucleotides fdC12L-S (5′-CAAAACCCGGGATAAATACGAGG-3′) (Sma I site underlined) and fdC12L-C (5′-GGCTGATCGATTGCGTGCTAC-3′) (Cla I site underlined), digested with Sma I and Cla I and inserted into the Sma I/Cla I-digested pGem-RG-fiC12L. The resulting plasmid pGem-RG-C12L was confirmed by DNA sequence analysis and directs the insertion of GFP gene into C12L locus of MVA-C.

### 3. Genetic modification of MVA

The deleted MVAΔC12L vector used in this work was generated using the clonal isolate MVA-F6, obtained after 582 passages in CEF cells [1], kindly provided by G. Sutter (Germany). Mutant MVAs were obtained following infection-transfection method previously described [53], employing monolayers of CEFs and the pΔC12L transference vector described above. MVAΔC12L was wt virus-free after six consecutive rounds of plaque purification on CEF cells in the presence of βGAL substrate (bluo-gal, halogenated indolyl-β-galactoside, Inalco, Italy). Purity of the selected clones was verified by PCR. MVAΔC12L-NefBF was generated using MVA-NefBF virus [36] and pΔC12L transference vector by infection/transfection method as described above, after six rounds of plaque purification. The MVAΔC12L-C vector was constructed by transient dominant selection using dsRed2 gene as the transiently selectable marker. MVAΔC12L-C was selected from progeny virus by consecutive rounds of plaque purification in DF-1 cells during which plaques were screened for Red2/GFP fluorescence. In the first three passages viruses from selected plaques expressed both fluorescent proteins while in the last three passages (six passages in total) viral progeny from selected plaques expressed only GFP due to the loss of dsRed2 marker.

### 4. In vitro characterization of MVAΔC12L viruses

The deletion of *008L* gene was confirmed by PCR using specific primers. Total DNA was extracted from uninfected or MVA and MVAΔC12L infected CEFs as described before [51]. The presence of *008L* and *165R* (coding for viral hemagglutinin) genes were evidenced by the amplification of a 1060 or 948 bp fragments using the following pairs of primers: 008i1/008d2 and HA1/HA4 [54], respectively. In order to evaluate the transcription of *008L* gene a RT-PCR was performed. Briefly, BHK-21 cell monolayers were uninfected or infected with MVA (wt o MVAΔC12L) at moi of 1. Twenty-four hs post inoculation (hpi) cells were harvested and RNA was extracted using Trizol®, according to the manufacturer instructions (Invitrogen, CA, USA). RNA samples were treated with DNase I (Invitrogen) and conserved at −70°C until used. RNA was reverse-transcribed with the reverse transcriptase M-MLVRT (200 U, Promega) using random hexamers. The cDNA obtained was used to amplify by PCR a fragment of 363 bp corresponding to the complete coding sequence for IL-18 bp. Primers used were 008L-F 5′ATGAAAATCCTATTTCTCATCGC and 008L-R 5′ CTACTTCAGCCAAATATTCT. To analyze kinetics of virus growth, CEFs monolayers grown in 60 mm tissue culture dishes were infected at 0.01 pfu/cell with MVAwt or MVAΔC12L. After 45 min of virus adsorption, the inoculum was removed, cells were washed twice and incubated with fresh medium plus 2% of FBS. For each virus and time-point (0; 6; 12; 16; 20; 24; 36; 48 and 72 h post-infection) cells (intracellular virus) and supernatants (extracellular virus) were collected separately, frozen/thawed three times and stored at −70°C until virus titers were determined by visualization of lysis plaques on CEFs. Each time point was evaluated by duplicate and each fraction was tittered twice.

### 5. Viral immunization stocks

Viral stocks were grown in BHK-21 cells (for MVA vectors) or BSC-40 cells (for VACV WR strain), viruses were released from the infected cells by several rounds of sonication, then purified by centrifugation through a sucrose cushion and titrated by immunostaining [55] in BHK-21 cells using a rabbit polyclonal antibody against VACV antigens (for MVA) or by plaque formation in BSC-40 cells using crystal violet (for WR).

### 6. IL-18-induced production of IFN-γ

Functional assays to evaluate the effects of *C12L* gene deletion on the IFN-γ production were performed as previously described [18]. Briefly, cultures of CEF cells were infected with MVAwt or MVAΔC12L at 5 pfu per cell. Supernatants from infected cells were harvested one day post-infection (dpi), centrifuged at 3000 rpm for 10 min at 4°C and the pellet was discarded. Residual viral particles were removed by centrifugation at 16.500 rpm for 60 min at 4°C and supernatants were stored at −20°C until use. Then, splenocytes from BALB/c naïve mice were cultured in RPMI 1640 medium plus 10% FBS (RPMIc) with 200 ng/ml concanavalin A (Con A) and 5 ng/ml murine IL-18 for 24 hs at 37°C. To test for inhibition of IL-18 action by the C12L protein, recombinant murine IL-18 was pre-incubated for 1 hr at room temperature with clarified supernatants from CEF cells that had been infected with the viruses. Finally, levels of IFN-γ in the culture medium were determined by a standard ELISA following the instruction of the manufacturer (BD Biosciences).

### 7. Immunization protocols, sample collection and processing

SPF BALB/c (*H-2d*) and C57BL/6 (*H-2b*) female mice, six to eight weeks old were purchased from the Laboratories of the School of Veterinary Sciences, University of La Plata, Buenos Aires, and then housed in our animal facilities. All experiments were carried out in strict accordance with the recommendations in the Guide for the Care and Use of Laboratory Animals of the National Institutes of Health [45]. The protocol was approved by the Committee of Care and Use of Laboratory Animals from the School of Medicine, University of Buenos Aires (Permit Number: 508/2009). Immunizations with viral vectors were given intraperitoneally (i.p) in 200 µl of PBS, intramusculary (i.m) in 200 µl of PBS (100 µl in each leg), subcutaneously (s.c) in 200 µl of PBS (100 µl in each leg, near to the groin region), or intranasally (i.n) in 20 µl of PBS (10 µl in each nasal fose). For DNA prime/MVA boost schemes, DNA doses were applied in 100 µl of sterile PBS by i.m route and 14 days after MVA boost was administered i.m or i.p. Doses and periods of time used in the different immunization schemes are depicted in the different Figures. Seven or forty days after the immunization, mice were sacrificed and after the recovery of spleens and draining lymph nodes (DLNs) under sterile conditions cells were isolated by standard procedures.

### 8. Analysis of specific T-cell immune responses

#### 8.1. Peptides for the evaluation of specific T-cell responses against vector epitopes

All VACV-specific synthetic peptides used in this work (8 or 15 aa long) were purchased from JPT Peptide Technologies (Germany). Lyophilized peptides were dissolved in dimethyl sulfoxide (DMSO) and stored at −20°C. All peptides were used at a final concentration of 2 µg/ml. E3 and F2(G) peptides have been previously described as CD8^+^ specific epitopes in BALB/c mice [56]; B8R (CD8^+^) and E9L, H3L, L4R (CD4^+^) are specific epitopes previously defined in C57BL/6 mice [28]. P815 (*H-2d* restricted) infected cells were used as APCs following the protocols described previously [57].

#### 8.2. Evaluation of specific T-cell responses against HIV-1 antigens

Overlapping synthetic peptides (13–15-mers, overlapping by 11 aa) were designed based on the Nef protein from CRF12_BF reference strain ARMA185, the same sequence employed for the construction of the DNA and MVA vectors that expressed this protein, and custom ordered from JPT Peptide Technologies (Germany). Overlapping synthetic peptides of NefB consensus protein were obtained from the NIH AIDS Reagent Program (catalog N° 9480). The peptides employed to evaluate the response against the HIV-1 subtype C antigens were previously described [39], they spanned the entire Env, Gag, Pol and Nef regions from clade C included in the immunogens as consecutive 15-mers overlapped by 11 amino acids. The CN54gp120 protein (499 aa) was spanned by the Env-1 (aa: 1–239; 49 peptides) and Env-2 (aa: 229–499; 63 peptides) pools. The Gag-Pol-Nef fusion protein (1417 aa) was spanned by the following pools: Gag-1 (aa: 1–254; 60 peptides), Gag-2 (aa: 244–500; 61 peptides),GPN-1 (aa: 485–735; 60 peptides), GPN-2 (aa: 725–831 and aa: 1017–1175; 61 peptides), GPN-3 (aa: 1165–1417; 61 peptides) and Nef (aa: 838–1044;49 peptides). Lyophilized peptides were dissolved in DMSO and stored at −20°C.

#### 8.2.1 Murine IFN-γ and IL-2 Elispot assays

Elispot assays were performed using freshly isolated splenocytes and cells from DLNs as previously described [24]. Briefly, 2×10^5^ to 10^6^ cells in RPMIc were plated in triplicate on nitrocellulose 96-well plates (MultiScreen HA plates; Millipore Corporation, Bedford) previously coated with an anti-mouse IFN-γ Ab (BD Pharmingen Rat anti-mouse IFN-γ, XMG1.2) or anti-mouse IL-2 Ab (BD ELISPOT Mouse IL-2 ELISPOT Pair). Stimulus consisted of VACV-specific individual peptides or overlapping synthetic peptides covering NefBF, NefB and different HIV-1 subtype C antigens as described above. To evaluate responses against P815-MVA infected cells, previously described protocols to infect the P815 *H-2d* MHC class I restricted cells, were followed [58]. Negative controls were incubated with RPMIc with the appropriate % of DMSO, and cells treated with Con A (1 µg/ml) were included as positive control.

The threshold values to consider a positive response by Elispot was that the number of specific spots/well had to be at least 2 times the average values found in negative control wells of each group. For evaluation of the response in the memory phase, the IFN-γ Elispot assay was performed using more sensitive anti-mouse IFN-γ Abs (BD ELISPOT Mouse IFN-γ ELISPOT Pair) which allowed the detection of subtle differences between vectors given the expected general reduction on the magnitude of the response at later times post-infection.

Functional avidity referred to as the activation threshold in response to defined concentrations of exogenous peptide was performed following the protocols previously described [59]. Briefly, limiting peptide dilutions (from 20 to 2×10^−6^ µg/ml) were performed and then the peptide concentrations needed to produce half-maximum IFN-γ production (number of spots) were calculated in *ex vivo* assays. Values of peptide concentrations needed for 50% of the maximal T-cell responses (EC50) were calculated with sigmoidal dose-response curves obtained with the GraphPad software.

#### 8.2.2 Simultaneous intracellular cytokine staining (ICS) and cytotoxic activity assessment

Splenocytes were dispensed in 96-well U bottom plates (2×10^6^ cells/well) and were stimulated with the VACV-specific peptides during 5 hs at 37°C in 5% CO_2_ in the presence of the costimulatory antibody anti-CD28 (1 ng/ml; BD Biosciences), brefeldin A (1 µl/ml GolgiPlug; BD Biosciences), monensin (0.7 µl/ml GolgiStop; BD Biosciences) and the monoclonal Abs (mAb) anti-CD107a and anti-CD107b both labeled with FitC (CD107a/b-FitC; BD biosciences; this molecules are degranulation markers, and allowed the detection of cytotoxic activity of CD8^+^ cells [60]). Negative and positive controls consisted of cells stimulated with RPMIc plus 0.08% DMSO, or PMA ionomycin (10 ng/ml phorbol myristate acetate [PMA] plus 250 ng/ml ionomycin [Sigma-Aldrich]) respectively). Afterwards, cells were washed and stained with surface antibodies (CD3-APC and CD8-PerCP; BD Biosciences) for 30 min at 4°C, and then permeabilized and fixed using the Cytofix/Cytoperm kit (BD Biosciences). After the permeabilization/fixation step, cells were stained using anti-tumor necrosis factor alpha (TNF-α) antibody labeled with PE-Cy7 (TNF-α-PE-Cy7; BD Biosciences) and anti-IFN-γ labeled with phycoerythrin (IFN-γ-PE; BD Biosciences) for 30 min at 4°C in obscurity, after two washes cells were stored at 4°C until being acquired in a BD FACSCanto flow cytometer. Data acquisition and analysis were done with the BD FACSDiva software. Instrument settings and fluorescence compensation were performed on each testing day using unstained and single-stained samples. Stimulated cells stained for surface molecules and isotype matched controls were included in each experiment.

#### 8.2.3 T-cell specific cytokine production

Splenocytes were suspended in RPMIc and cultured in triplicate (10^6^ cells/well) into 96-well microtiter flat-bottom plates and stimulated with the indicated peptides. Positive and negative controls were cells stimulated with Con A (1 µg/ml), and with medium plus the appropriate % of DMSO respectively. After 72 hr incubation at 37°C in 5% CO_2_, culture supernatants were harvested at −80°C and analyzed by ELISA for IFN-γ (BD OptEIA Set Mouse IFN-γ) and TNF-α (BD OptEIA Set Mouse TNF mono/poly) following the manufacturer's instructions.

### 9. Intranasal challenge with replication competent WR

Groups of eleven BALB/c mice were vaccinated with 10^6^ pfu of MVAwt, MVAΔC12L or mock inoculated by the i.p route. At 45 days post-immunization, animals were anesthetized by i.p injection of ketamine (0,1 mg/g) and xylazine (0,01 mg/g), infected i.n with Vaccinia virus WR (2×10^6^ pfu diluted in 20 µl PBS, 10 µl in each nasal fose) and monitored for at least 12 days for morbidity and mortality, with daily measurement of individual body weights and observation of signs of illness as described previously [52]. In brief, signs of illness recorded were: bristly fur curved back, difficulty to breathe, reduced motility, and corporal tremors; they were measured by the assignment of a score proportional to the number of signs present in each mouse.

Animals suffering from severe systemic infection and having lost >30% body weight were sacrificed (humane end point). The % of accumulative body weight loss was calculated as the relative % weight loss registered each day with respect to the immediate previous day plus the % weight loss accumulative registered until that moment. Thus, the % of accumulative weight (W) loss registered at n day was calculated with the following formula:
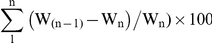



### 10. Data analysis

All data were expressed as the mean ± SD of triplicate (Elispot) and duplicate (ELISA and ICS) determinations for each group, and are representative of at least three independent experiments. The significance of differences between the different groups of immunized mice was determined using the two-tailed Student's *t* test or the Mann Withney test (GraphPad Prism4 software). Values of p≤0.05 were considered statistically significant.

## Supporting Information

Figure S1 **Mice vaccinated with MVAΔC12L show less signs of illness after a VACV challenge.** The three groups of BALB/c mice used in the VACV challenge (see legend Fig. 6C) were also monitored for signs of illness appearance as described in Materials and Methods. The figure shows the mean score ± SD for each group from days 5 to 8 at which differences in weight loss were substantial (see Fig. 6C and D). The asterisks represent the statistically significant differences between MVAwt vs. MVAΔC12L (** p<0.01, *** p<0.001). Statistically significant differences between mock-vaccinated vs. both MVAwt and MVAΔC12L groups: ★ p<0.001.
(TIF)
