# Supplementary figures and images for: Improving the MVA Vaccine Potential by Deleting the Viral Gene Coding for the IL-18 Binding Protein
Source: PLoS One. 2012 Feb 22;7(2):e32220. doi: 10.1371/journal.pone.0032220 (PMC3285208; doi:10.1371/journal.pone.0032220)

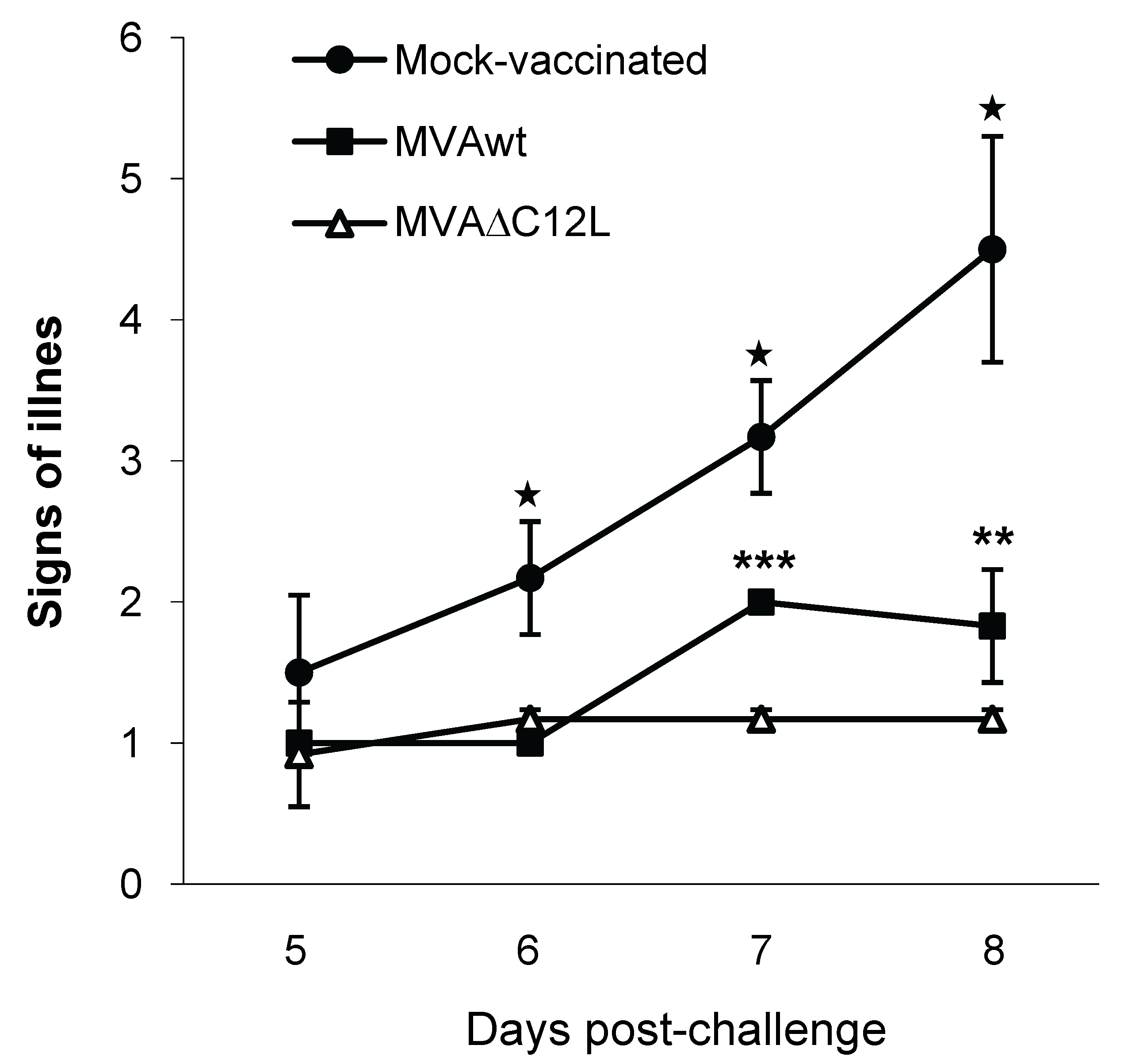

Supplement: Figure S1 — Mice vaccinated with MVAΔC12L show less signs of illness after a VACV challenge. The three groups of BALB/c mice used in the VACV challenge (see legend Fig. 6C) were also monitored for signs of illness appearance as described in Materials and Methods. The figure shows the mean score ± SD for each group from days 5 to 8 at which differences in weight loss were substantial (see Fig. 6C and D). The asterisks represent the statistically significant differences between MVAwt vs. MVAΔC12L (** p<0.01, *** p<0.001). Statistically significant differences between mock-vaccinated vs. both MVAwt and MVAΔC12L groups: ★ p<0.001. (TIF) [file pone.0032220.s001.tif]
